# Supplementary material for: Cysteamine/Cystamine Exert Anti-Mycobacterium abscessus Activity Alone or in Combination with Amikacin
Source: Int J Mol Sci. 2023 Jan 7;24(2):1203. doi: 10.3390/ijms24021203 (PMC9866335; doi:10.3390/ijms24021203)
Supplement: Supplementary file 1 [file ijms-24-01203-s001.zip › ijms-2101229-supplementary-Figure S1.PDF]

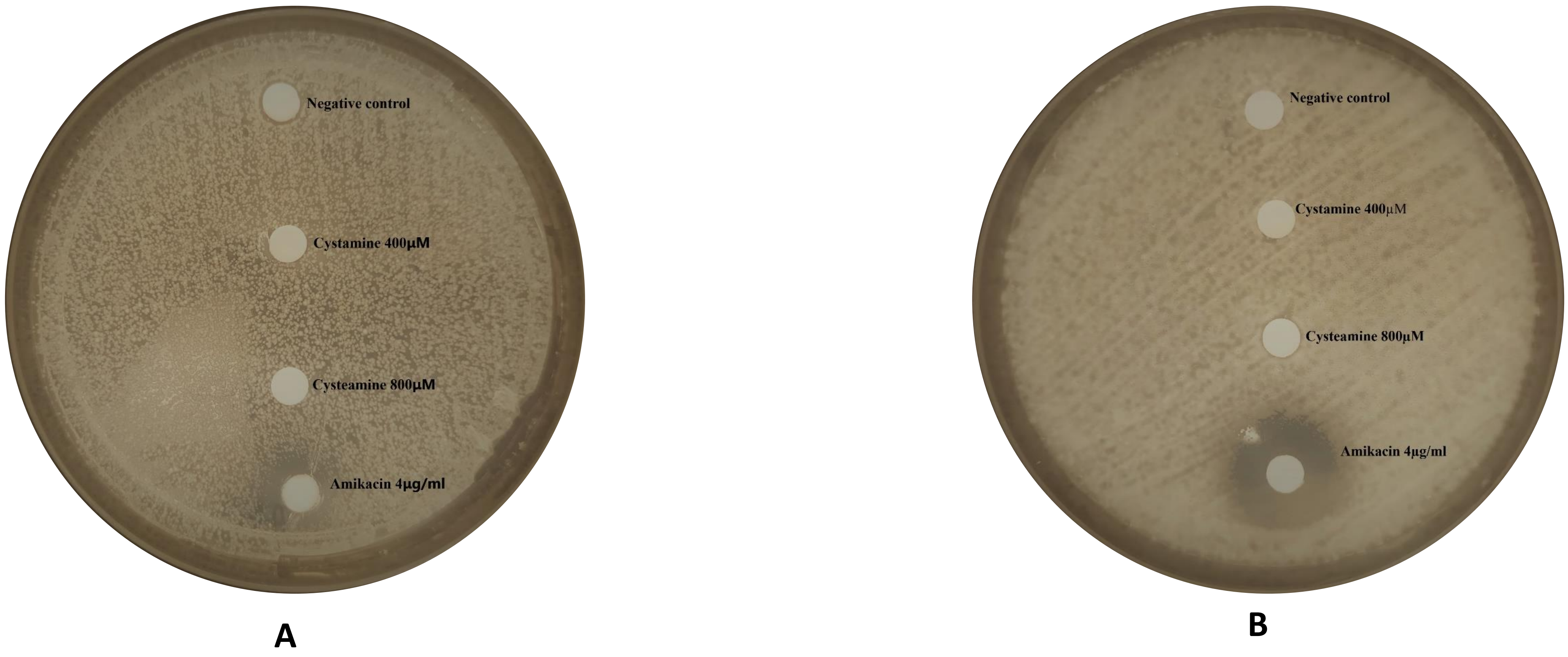

Supplementary Figure S1: Inhibition of bacterial growth by spotting assay. Each *M. abscessus* variant (MAB-S in **A** and MAB-R in **B**) was grown on 7H11 agar plates and spots previously soaked for few hours in solutions containing the drugs under study at the concentrations indicated in the pictures. Spots soaked in 7H9 was used as a negative control. Plates were incubated at 37 °C for three days.
