# Supplementary material for: Cysteamine/Cystamine Exert Anti-Mycobacterium abscessus Activity Alone or in Combination with Amikacin
Source: Int J Mol Sci. 2023 Jan 7;24(2):1203. doi: 10.3390/ijms24021203 (PMC9866335; doi:10.3390/ijms24021203)
Supplement: Supplementary file 1 [file ijms-24-01203-s001.zip › ijms-2101229-supplementary-Figure S2.PDF]

Supplementary Figure S2

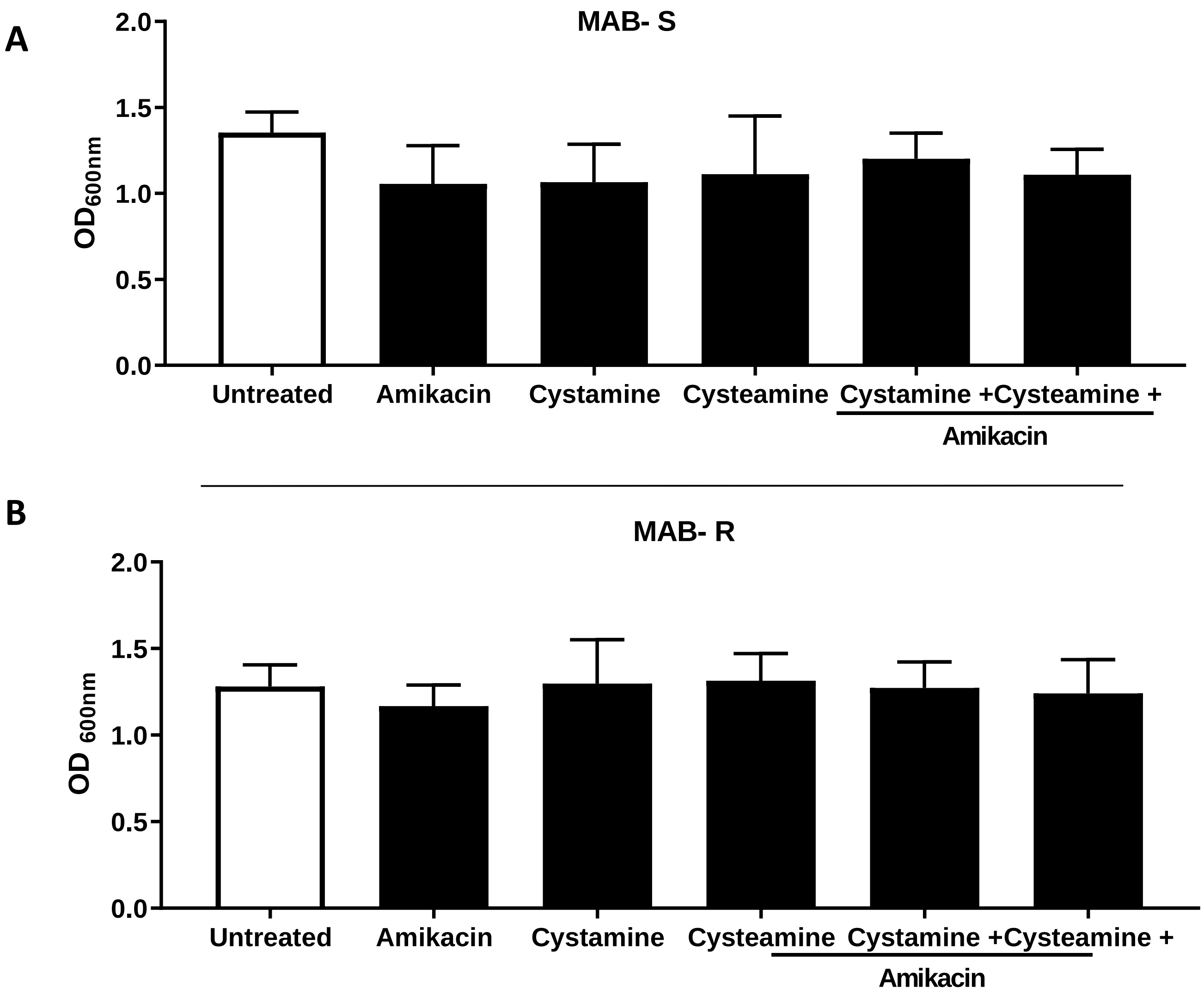

Supplementary Figure S2: LDH cytotoxicity assay: Supernatants from PBMCs infected with *M. abscessus* S (**A**) and R (**B**) variants were assayed for LDH to determine cytotoxicity. The results are presented as the mean ± standard deviation of OD of three independent experiments. No statistically significant differences were observed between groups. # LDH, lactate dehydrogenase.
