# Supplementary material for: Cysteamine/Cystamine Exert Anti-Mycobacterium abscessus Activity Alone or in Combination with Amikacin
Source: Int J Mol Sci. 2023 Jan 7;24(2):1203. doi: 10.3390/ijms24021203 (PMC9866335; doi:10.3390/ijms24021203)
Supplement: Supplementary file 1 [file ijms-24-01203-s001.zip › ijms-2101229-supplementary-Figure S3.PDF]

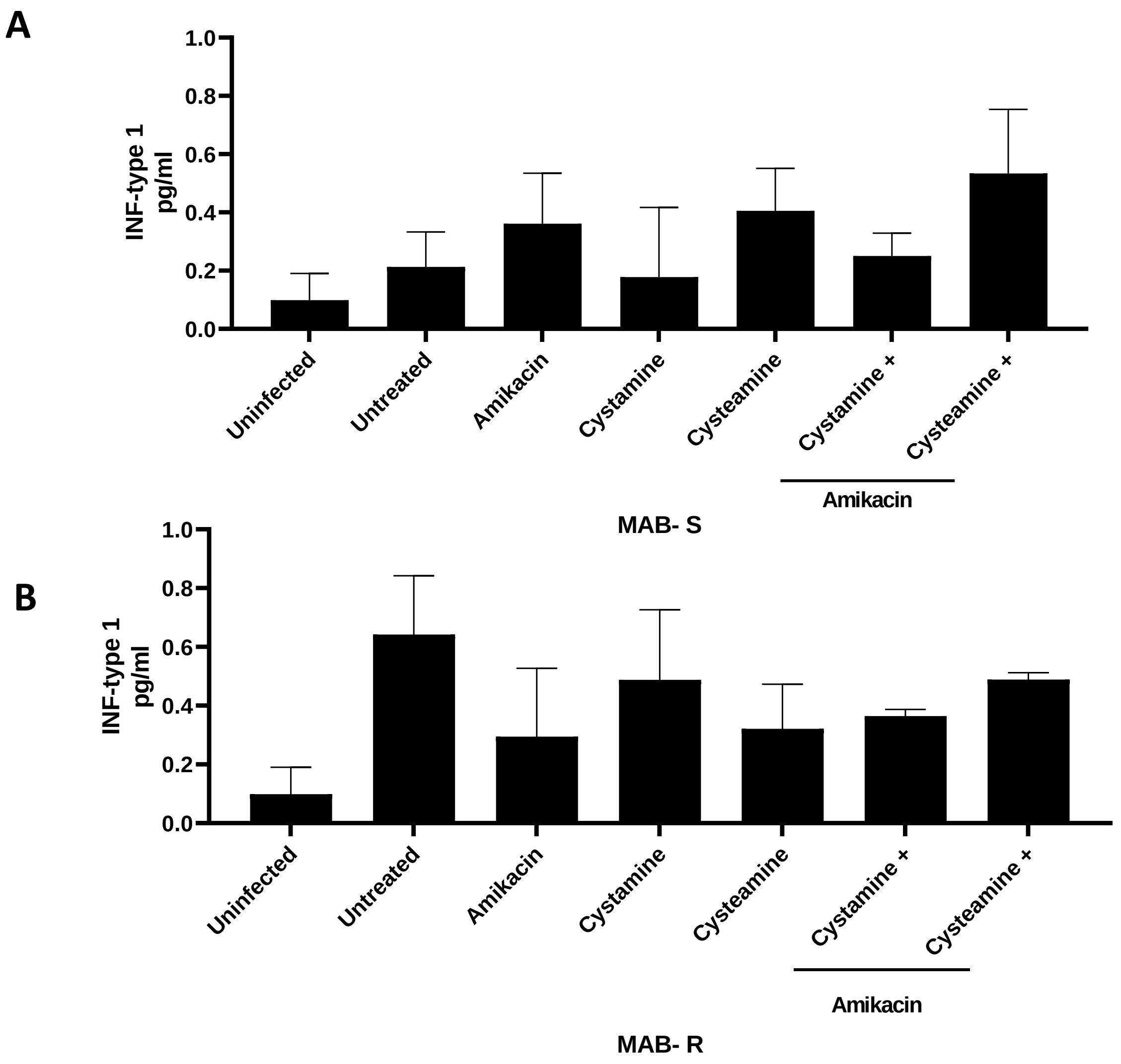

Supplementary Figure S3: Evaluation of Type I IFN: HEK-blue α/β cells were treated with supernatants of PBMCs infected with MAB-S **(A)** and MAB-R **(B)** strains strain at 12 days p.i. and production of SEAP in culture medium was determined and measured at 630 nm optical density (O.D.). Data are expressed as mean ± standard deviation of OD for at least two independent experiments. P- values were determined One ANOVA Dunnett's multiple comparisons test and no statistically significant differences were observed between groups.
